# QT Prolongation Preceding Ventricular Fibrillation After Amiodarone Administration: A Case Report

**DOI:** 10.7759/cureus.63763

**Published:** 2024-07-03

**Authors:** Milovan Stojanovic, Miroslav Nikolic, Ivana Nedeljkovic, Dejana Gojkovic

**Affiliations:** 1 Department for Cardiovascular Diseases, Institute for Treatment and Rehabilitation Niska Banja, Nis, SRB; 2 Faculty of Medicine, Nis University, Nis, SRB; 3 Clinic for Cardiovascular Diseases, University Clinical Center Nis, Nis, SRB; 4 Clinic for Cardiovascular Diseases, University Clinical Center of Serbia, Belgrade, SRB; 5 Faculty of Medicine, Belgrade University, Belgrade, SRB

**Keywords:** induction of ventricular fibrillation, qt interval prolongation, torsades de pointes (tdp), intravenous amiodarone, atrial fibrillation (af)

## Abstract

Atrial fibrillation (AF) is the most common long-term arrhythmia in adults. Rhythm control in patients with AF involves efforts to restore and maintain sinus rhythm and is accomplished by medication, catheter ablation, or electrical cardioversion. Amiodarone represents one of the most commonly used antiarrhythmic medications. Prolonged use of amiodarone can lead to many side effects. One of the most severe side effects is drug-induced long QT syndrome (LQTS), which can cause malignant arrhythmias and sudden cardiac death. We presented a case of a 52-year-old male who was admitted to the Coronary Unit due to first diagnosed AF with a rapid ventricular response. After amiodarone infusion was administrated the patient lost consciousness and the monitor displayed torsades de pointes (TdP) ventricular tachycardia with rapid conversion to ventricular fibrillation (VF). Cardiac resuscitation with two direct current (DC) shocks was performed. The patient was stabilized, and restoration of sinus rhythm with significant QT prolongation on the ECG was noted. This is a rare case of short-term amiodarone administration causing LQTS, TdP, and VF. The findings or observations emphasize the significance of diligent ECG monitoring during amiodarone treatment.

## Introduction

Atrial fibrillation (AF) is the most common long-term arrhythmia in adults with a prevalence of between 2% and 4%, and is a risk factor for stroke, heart failure, premature death, and cognitive impairment [1]. The pathogenesis of AF is complex and includes some common risk factors such as gender (more prevalent in males), age (increases with age), ethnicity (more frequent in Caucasians), and genetics [1]. However, there is a wide range of modifiable factors (arterial hypertension, endocrine disorders, chronic obstructive pulmonary disease, obesity, etc.) whose treatment could influence the occurrence of AF.

The adequate treatment of AF is based on the ABC pathway: "A" - anticoagulant therapy to prevent thromboembolic events and avoid stroke; "B" - chasing rate/rhythm control to reduce symptoms; and "C" - treating comorbidities [1]. Better symptom control can be achieved by rate or rhythm control. Rhythm control involves efforts to restore and maintain sinus rhythm and is accomplished by medication, catheter ablation, or electrical cardioversion. Amiodarone represents one of the most commonly used antiarrhythmic medications [2].

Although it affects all phases of the cardiac action potential, amiodarone is classified as a class III antiarrhythmic drug commonly used for the treatment of supraventricular and ventricular arrhythmias, usually in patients with known or suspected structural heart disease [3]. However, prolonged administration of amiodarone can lead to many serious side effects. For example, in about 1-2% of patients, amiodarone causes lung fibrosis and hypersensitivity - complications that have a fatal outcome in approximately 10% of cases [4]. Additionally, although fatal hepatic failure is uncommon, about 1% of patients develop a mild increase in transaminases [4]. Amiodarone-induced hypothyroidism and hyperthyroidism are commonly seen in everyday clinical practice [5].

Prolonged use of amiodarone can lead to drug-induced long QT syndrome (LQTS). LQTS is defined as a heart rate-corrected QT interval (QTc) ≥ 480 ms or a “Schwartz” score higher than 3 points [6]. LQTS can lead to malignant arrhythmias and sudden cardiac death (SCD) [7]. Amiodarone is a prodrug and needs to be metabolized in the liver. There are three phases of distribution during amiodarone loading: vascular or central distribution (first 24 hours), distribution in organs (next seven days), and distribution in adipose tissue (approximately the next four weeks). It takes more than two months for amiodarone to reach its full antiarrhythmic effects. This is why amiodarone side effects usually occur after prolonged administration. However, the effect of short-term administration of amiodarone on the QT interval has not been established.

## Case presentation

**Figure 1:**
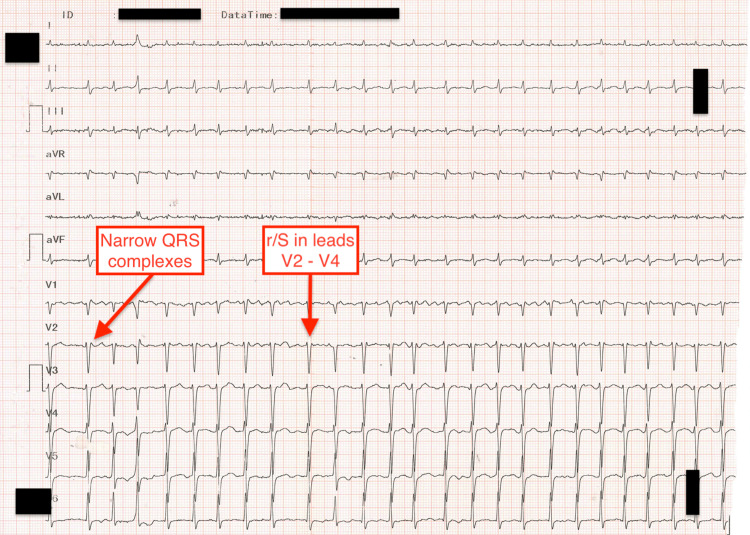
Electrocardiogram at the admission to the Emergency Department: atrial fibrillation, normal axis, heart rate of around 150/min, narrow QRS complexes, and r/S in leads V2-V4

**Figure 2:**
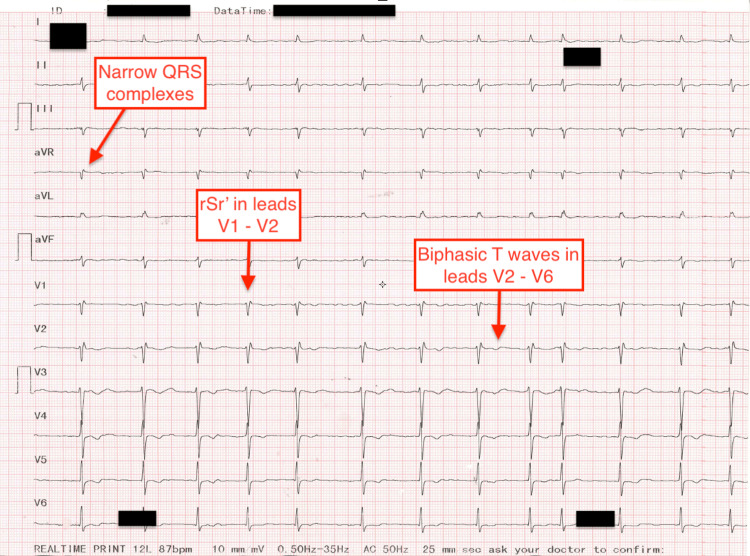
Electrocardiogram after medical therapy and slowing down the heart rate: atrial fibrillation, left axis, heart rate of around 80/min, narrow QRS complexes, rSr' in leads V1 and V2, and biphasic T waves in leads V2-V6

A 52-year-old male was admitted to the Emergency Department of the Institute for Treatment and Rehabilitation "Niška Banja" due to dyspnea and palpitations. An electrocardiogram (ECG) revealed AF with rapid ventricular response (Figure 1). The onset of symptoms was two hours before the examination, and it was established that, apart from antihypertensive therapy (ramipril 5 mg once a day, and nebivolol 2.5 mg once a day), he had not taken any medication until then. The CHA2DS2VASc score was 1. Low-molecular-weight heparin (1 mg/kg subcutaneously) was administered, and his heart rate was reduced to 80 bpm with IV digoxin 0.25 mg, IV metoprolol 5 mL, and IV amiodarone 150 mg (Figure 2). The patient was feeling better. After decreasing his heart rate, an echocardiography was performed, and the dilatation of the left ventricle (end-diastolic diameter of 62 mm) and fibrosis of the interventricular septum (IVS) were noticed. The ejection fraction was preserved (EF 52%), and the left atrium was dilated (47 mm) (Table 1).

**Table 1:** Parameters of cardiac ultrasound LV – left ventricle

Variables	Values	Normal range
End-diastolic diameter of LV (mm)	62	39-58
End-systolic diameter of LV (mm)	45	24-40
Fractional shortening (%)	27	24-42
Ejection fraction (%)	52	≥52
Interventricular septum (mm)	10	60-10
Posterior wall (mm)	10	6-10
Right ventricle (mm)	24	≤28
Aortic root (mm)	39	<32
Left atrium (mm)	47	<40

The patient was then admitted to the Coronary Care Unit where laboratory assessment was done. Troponin levels were in the normal range, as well as the inflammatory markers (C-reactive protein of 1.6 mg/dL) and electrolytes (potassium of 4.2 mmol/L, sodium of 140 mmol/L) (Table 2). Since this was the first episode of AF in a relatively young patient with no comorbidities, it was decided that sinus rhythm should be restored. Because of the dilatation of the left ventricle and fibrosis of the IVS, it was chosen to continue with an amiodarone infusion.

**Table 2:** Laboratory measurements hs - highly sensitive

	I	II	III	IV	V	VI	VII	VIII	IX
Glucose (mmol/L)		5.5			8.2	6.3	6.4	7.2	4.3
Creatinine (µmol/L)		93.3	93.1	81.3	78.9	84.8	76.8	79.5	78.3
Uric acid (µmol/L)		411							
Urea (mmol/L)		5.2		5.8	4.2	7.9	10.5	13.3	
Proteins (g/L)		63						64	
Cholesterol (mmol/L)		3						5.43	4.51
C reactive protein (mg/L)	1.6	47.7	29.6			98	20.4		
Magnesium (mmol/L)			1.4	0.9	0.73	0.99			
Calcium (mmol/L)		2.23			2.26			6.9	1.2
Albumin (g/L)		38			40				
Potassium (mmol/L)	4.2	3.6	4.6	4.4	3.6	3.6	3.8	4	4.1
Sodium (mmol/L)	140	140	141	140	137	140	139	136	137
Aspartate aminotransferase (U/L)		77			95			21	29
Alanine transaminase (U/L)		90			120			31	31
hsTroponin (ng/mL)		0.03	0.02					0.059	
Brain natriuretic protein (pg/mL)		505		411				54	
Thyroid-stimulating hormone (mIU/L)					2.197				0.397
D dimer (ng/mL)					13659				
Troponin (ng/mL)	0.01								

**Figure 3:**
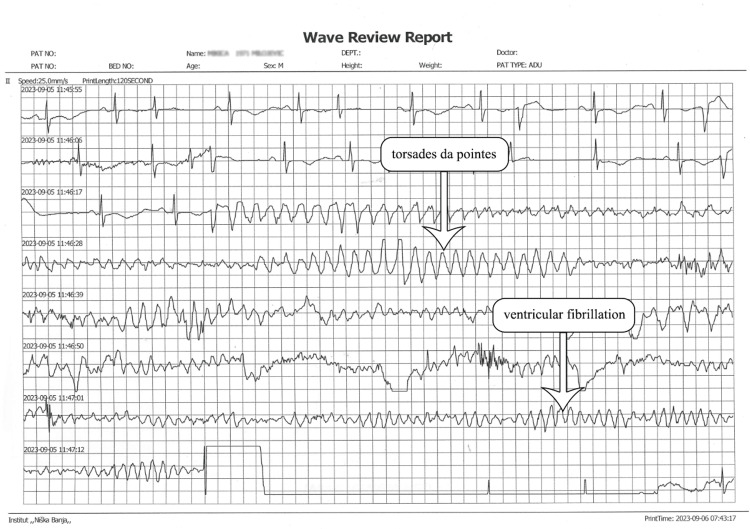
Electrocardiogram after amiodarone infusion: sinus rhythm and then torsades de pointes with rapid conversion to ventricular fibrillation

**Figure 4:**
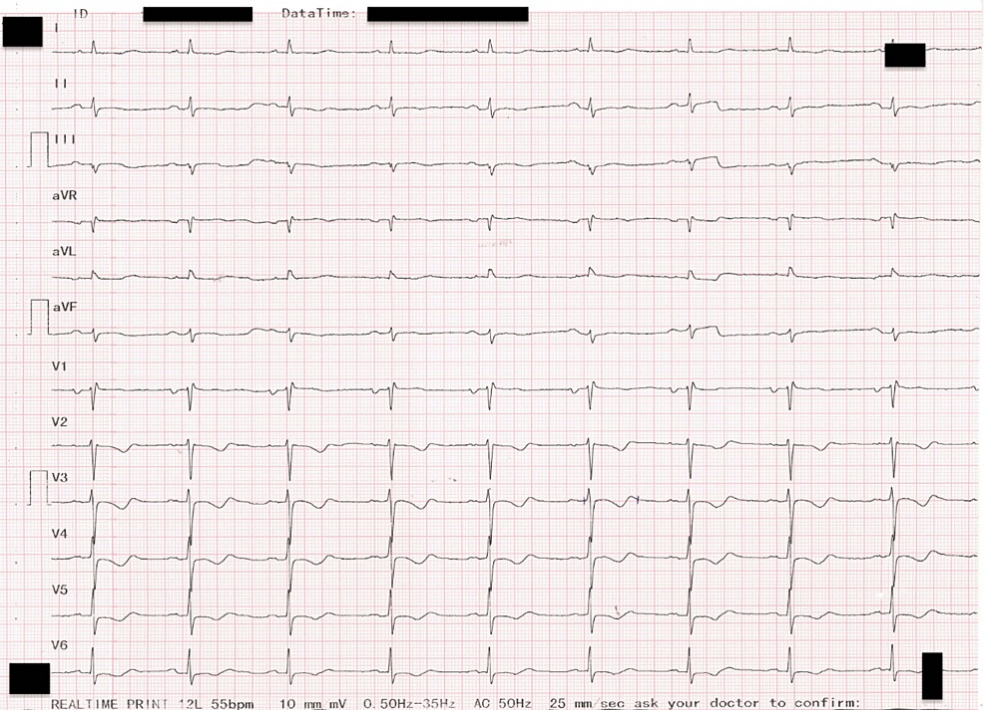
Electrocardiogram after DC shock: sinus rhythm, left axis, heart rate of 52/min, PR interval of 0.20 seconds, narrow QRS complexes, rSr' in V2 and V3, biphasic T waves in V2-V6, and prolonged QT interval

The infusion of 250 mL of 5% glucose saline and two ampoules of amiodarone (one ampule containing 3 mL/150 mg of amiodarone) was initiated. After a couple of hours, the patient lost consciousness, and the monitor displayed torsades de pointes (TdP) ventricular tachycardia with rapid conversion to ventricular fibrillation (VF) (Figure 3). Cardiac resuscitation was started, and electrical cardioversion with two direct current (DC) shocks was performed. The patient was stabilized, and sinus rhythm was restored with biphasic T waves in leads V2-V6 and significant QT prolongation (QTc=541 ms, calculated by Bazett's formula [8]) on the ECG (Figure 4).

**Figure 5:**
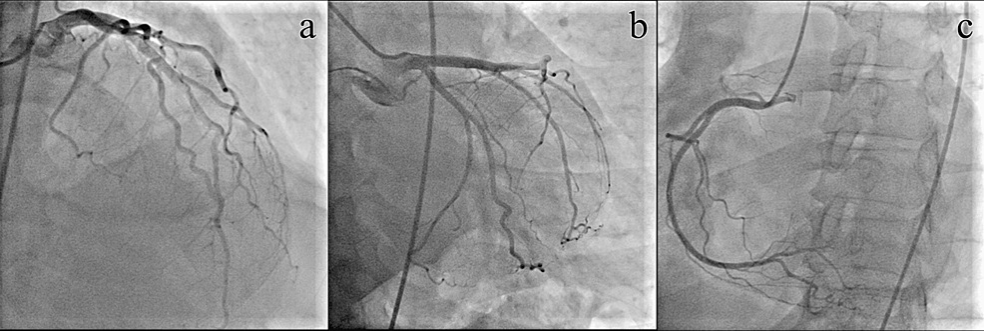
Left coronary angiogram in cranial right anterior oblique (c-RAO) (a) and caudal view (b) and coronary angiogram of the right coronary artery (RCA) in the left anterior oblique view (c) showing no obstructive stenosis

The amiodarone infusion was stopped, and beta-blocker (metoprolol 2 mL IV) and magnesium infusions (2 mg IV) were introduced. Shortly after, another episode of VF occurred and was terminated by a new electrical cardioversion. The infusion of lidocaine (1 mg/kg) was initiated, and the patient was transferred to the Clinic for Cardiovascular Disease, University Clinical Center Nis, for coronary angiography, which did not reveal any significant stenotic lesions (Figure 5). Repeated troponin analyses were within the normal range. Cardiac magnetic resonance (CMR) was performed. It did not show any scar tissue, fibrosis, or signs of acute myocardial inflammation. The left ventricle was dilated (EDD 61 mm), and the ejection fraction was reduced (EF 38%).

**Figure 6:**
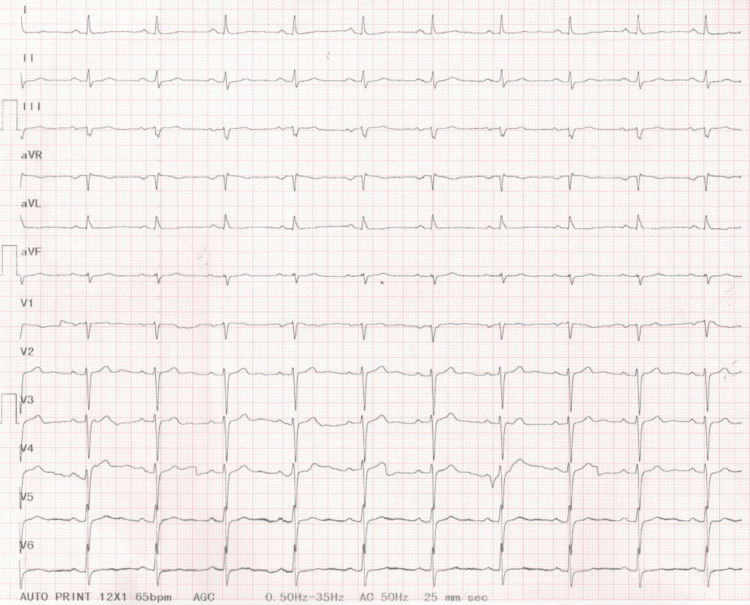
Electrocardiogram at the discharge from hospital: sinus rhythm, left axis, heart rate of 62/min, PR interval of 0.20 seconds, narrow QRS complexes, and r/S in V2-V4

During hospitalization, the QTc interval decreased to 438 ms with T-wave positivity (Figure 6). The patient was hemodynamically and rhythmically stable, and episodes of VF did not occur again. However, a cardioverter defibrillator was implanted as a secondary prevention of sudden cardiac death. The patient was discharged with the following therapy: bisoprolol 2.5 mg, mexiletine 2x200 mg, rivaroxaban 15 mg, spironolactone 25 mg, dapagliflozin 10 mg, and pantoprazole 20 mg.

## Discussion

Our case showed an amiodarone-induced prolongation of the QT interval that led to TdP and VF. QT interval should be an integral part of ECG evaluation, as the prolongation of QT can lead to malignant arrhythmias and SCD [7]. It is measured from the beginning of the QRS complex to the end of the T wave and is usually corrected for heart rate according to Bazett's or Fridericia's formula. QT interval incorporates both ventricular depolarization (QRS complex) and repolarization (J-T interval). This means that any problem with depolarization (fast inward sodium current) or repolarization (inward calcium and sodium current, outward potassium current) can prolong QT. Acquired LQTS is far more common in everyday clinical practice when compared to inherited ones [9]. Many drugs, such as antiarrhythmics, antibiotics, antihistamines, and antipsychotics, are known to prolong the QT interval [9].

Amiodarone is a class III antiarrhythmic agent that blocks potassium channels and prolongs the duration of action potential. In this way, amiodarone increases the refractory period of atrial and ventricular tissues. Although remarkably efficacious in the treatment of ventricular and atrial arrhythmias, amiodarone has many side effects, whose prevalence increases with the duration of therapy [4]. One of the most serious side effects of amiodarone is QT interval prolongation, as it can cause polymorphic ventricular tachycardia that can lead to VF and SCD. Amiodarone-induced QT interval prolongation is caused by potassium channel blockage, which causes the prolongation of ventricular repolarization and refractory period [10].

According to the literature, the incidence of amiodarone-induced TdP is less than 1% [11], and it increases with IV administration [12]. TdP usually occurs within 24 hours after initiation of the IV therapy [12], but it can happen even three days after amiodarone infusion [11]. However, it is extremely rare for short-time administration of amiodarone to cause LQTS and TdP [11-13]. The risk factors for malignant arrhythmias in amiodarone-induced LQTS are rapid amiodarone administration, renal failure, acute myocardial infarction, heart failure, hypokalemia, hypomagnesemia, genetic predisposition, bradycardia, QTc interval >500 ms and/or QTc interval increase by ≥60 ms from the baseline value [13]. The highest risk for malignant arrhythmias occurrence is 6-24 h after IV administration of amiodarone [12]. This is why the monitoring of ECG during the first 24-48 hours of IV amiodarone infusion is suggested.

The amiodarone-induced LQTS demands immediate discontinuation of amiodarone and, if TdP occurs, magnesium and lidocaine administration. Furthermore, it is crucial to investigate and treat any other risk factors that may potentiate the pro-arrhythmogenic effects of amiodarone. For example, hypomagnesemia or hypokalemia increases the risk of amiodarone-induced TdP [12]. If recognized and treated early, patients with amiodarone-induced TdP have a good prognosis. However, amiodarone should not be reinitiated in these patients under any circumstances [12].

In our patient, coronary angiography was performed, and coronary artery disease was excluded. Moreover, arrhythmogenic cardiomyopathy and myo- and pericarditis were excluded by normal MRI. Troponin, natriuretic, and electrolytes were consistently monitored and remained within the normal range, except after DC shock was performed. This is why we believe that short-term administration of amiodarone probably led to QT prolongation, which caused TdP and VF. After amiodarone was discontinued, QTc normalized and arrhythmia ceased. However, we opted to implant a cardiac defibrillator for the secondary prevention of SCD.

## Conclusions

Although remarkably efficacious in the treatment of ventricular and atrial arrhythmias, amiodarone has many side effects, whose prevalence increases with the duration of therapy. One of the most severe side effects is drug-induced LQTS, which can cause malignant arrhythmias and sudden cardiac death.

We presented a case of a 52-year-old male patient with amiodarone-induced LQTS, which led to TdP tachycardia with rapid conversion to ventricular fibrillation who needed cardiac resuscitation. This case reiterates the importance of remembering that anti-arrhythmics, particularly class III (potassium channel blockers), are also pro-arrhythmic with the possibility of immediate fatal consequences. By delaying repolarization and increasing the action potential duration, the QTc interval is increased, and there is an elevated risk of developing TdP early after depolarizations (EAD). Although the incidence of amiodarone‐induced TdP is <1% and is more common in other class III drugs, such as sotalol and ibutilide, this case further illustrates the point that anti-arrhythmic drugs are also pro-arrhythmic. This case also demonstrates the importance of looking at a patient's complete picture rather than reflexively resorting to an anti-arrhythmic drug for ventricular arrhythmias.
